# Adaptive mechanism of *Lactobacillus amylolyticus* L6 in soymilk environment based on metabolism of nutrients and related gene‐expression profiles

**DOI:** 10.1002/fsn3.2779

**Published:** 2022-02-23

**Authors:** Yongtao Fei, Li Huang, Hong Wang, Jinglong Liang, Gongliang Liu, Weidong Bai

**Affiliations:** ^1^ 47894 Guangdong Provincial Key Laboratory of Lingnan Specialty Food Science and Technology Zhongkai University of Agriculture and Engineering Guangzhou China; ^2^ 47894 College of Light Industry and Food Science Zhongkai University of Agriculture and Engineering Guangzhou China; ^3^ 47894 Academy of Contemporary Agricultural Engineering Innovations Zhongkai University of Agriculture and Engineering Guangzhou China

**Keywords:** chemical components, *Lactobacillus amylolyticus* L6, soymilk, transcriptome

## Abstract

*Lactobacillus amylolyticus* L6 isolated from naturally fermented tofu‐whey was characterized as potential probiotics. To give insight into the adaptive mechanism of *L. amylolyticus* L6 in soymilk, the gene‐expression profiles of this strain and changes of chemical components in fermented soymilk were investigated. The viable counts of *L. amylolyticus* L6 in soymilk reached 10^12^ CFU/mL in the stationary phase (10 hr). The main sugars reduced gradually while the acidity value significantly increased from 45.33° to 95.88° during fermentation. About 50 genes involved in sugar metabolization and lactic acid production were highly induced during soymilk fermentation. The concentration of total amino acid increased to 668.38 mg/L in the logarithmic phase, and 45 differentially expressed genes (DEGs) in terms of nitrogen metabolism and biosynthesis of amino acid were detected. Other genes related to lipid metabolism, inorganic ion transport, and stress response were also highly induced. Besides, the concentration of isoflavone aglycones with high bioactivity increased from 14.51 mg/L to 36.09 mg/L during the fermentation, and the expression of 6‐phospho‐*β*‐glucosidase gene was also synchronously induced. This study revealed the adaptive mechanism of *L. amylolyticus* L6 in the soymilk‐based ecosystem, which gives the theoretical guidance for the application of this strain in other soybean‐derived products.

## Graphical abstract

During fermentation, the main sugars reduced gradually while the acidity value significantly increased from 45.33° to 95.88° during fermentation. About 50 genes involved in sugar metabolism and lactic acid production were highly induced during soymilk fermentation. The concentration of total amino acid increased to 668.38 mg/L in the logarithmic phase, and 45 differentially expressed genes (DEGs) in terms of nitrogen metabolism and biosynthesis of amino acid were detected.

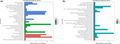

## 1 INTRODUCTION

Soybean and its derived food products are important part of the Asian diet. These foods are rich in various nutrients, such as protein, oligosaccharides (raffinose and stachyose), grease, vitamins, and insoluble dietary fiber (Lokuruka, 2011). Meanwhile, they were reported to have many beneficial functions to consumers, including the prevention of cardiovascular disease, osteoporosis, hormone‐related cancers, and modulation of immunity and intestinal flora (Ko, 2014; Yan et al., 2017). As consumers become increasingly interested in functional foods, they have higher requirements for the varieties of functional soybean products. In recent years, fermentation of soymilk by probiotics has become one of the research hotspots because of the function‐promoting effects brought about by these microorganisms and soymilk (Marazza et al., 2013; Wang et al., 2012; Wei et al., 2007).

Probiotics were able to attach to the surface of intestinal mucosa and colonize in the intestinal tract, which could allow them to bring beneficial effects to human health (Bron et al., 2012). For example, the probiotics could competitively exclude and inhibit pathogens in the intestinal tract (Kholy et al., 2014), enhance intestinal flora (Gerritsen et al., 2011), augment both cellular and humoral immunity (Yan & Polk, 2011), and relieve inflammation and food allergy (Majamaa & Isolauri, 1997). Except for the above functional characteristics, the probiotics used as soymilk starter were required to adapt to a complex nutritional environment of soymilk. In general, the minimum number of living probiotics in the final product of soybean yoghurt should reach 10^8^ CFU/ml (Shah, 2000). Meanwhile, the pH for coagulating soymilk was 4.5–5.0 (Qiao & Li, 2007), which required the strong acid‐producing ability of probiotics in soymilk. Although the stachyose and raffinose in soymilk have been regarded as prebiotics, excessive intake by human body would cause gastric bloating or diarrhea, requiring probiotics to own ability of hydrolyzing soybean oligosaccharides in soymilk with α‐galactosidase (Donkor et al., 2007). In addition, soymilk is rich in low‐absorptive isoflavone glycosides (occupying approximately 90% of isoflavone content) (Izumi et al., 2000), and the probiotic strain with the ability of converting isoflavone glycosides into high‐absorptive aglycones by *β*‐glucosidase were the best choice (Donkor et al., 2007). On the other hand, stachyose and raffinose in soymilk could promote the proliferation of fermentation probiotic strains (Kim et al., 2010; Sarina et al., 2017). In addition, the soymilk could be used as food vehicles of probiotics, protecting bacterial cells from adverse environment such as low pH of gastric acid, bile salt, and various digestive enzymes in the gastrointestinal tract (Zhuang et al., 2009). Therefore, the selection of probiotic strains suitable for soymilk environment is very important for the production of soybean yoghurt.

*Lactobacillus amylolyticus* L6 was isolated from naturally fermented tofu‐whey, a traditional Chinese tofu‐coagulant (Fei et al., 2018), and its safety, potential probiotic characteristics, and fermentation properties in tofu‐whey have been extensively studied (Fei et al., 2020; Fei, Li, et al., 2017; Fei, Liu, et al., 2017). Since *L. amylolyticus* L6 was one of the dominant bacteria in naturally fermented tofu‐whey for a long time, it has evolved the adaptability to nutritional environment in soybean products, which makes *L. amylolyticus* L6 one of the best candidate probiotic strains for fermenting soymilk. In this study, the changes of nutrient and functional substances in soymilk and gene‐expression profiles of *L. amylolyticus* L6 during fermentation were investigated to reveal the molecular mechanisms of synergistic effect between soymilk and *L. amylolyticus* L6.

## 2 MATERIALS AND METHODS

### 2.1 Strains and cultivation

*Lactobacillus amylolyticus* L6 (CGMCC NO.9090) was isolated from naturally fermented tofu‐whey (Fei et al., 2018). This strain was preserved in 15% glycerol at −80°C and cultivated in De Man, Rogosa and Sharpe (MRS) (Guangdong Huankai Microbiology Biotech Inc., Guangzhou, China) plate at 37°C for 36 hr before use. A single colony was then picked and inoculated into 10 ml of MRS broth and incubated for 24 hr.

### 2.2 Preparation of fermented soymilk

Soymilk was prepared according to the method described by Salma et al. (Elghali et al., 2014) with slight modification. Soybean (100 g) was washed and then soaked in 600 ml of drinking water with 0.5% NaHCO_3_ at 26°C for 14 hr. The soaked soybean was ground and heated with 800 ml of drinking water in a soymilk maker (DJ12B‐DEF4, Midea, China). The slurry was filtered through a double‐layered cotton cloth and then mixed with drinking water in a ratio of 8:2. Glucose (Sigma Chemical Co., Ltd, Guangzhou, China) with a concentration of 1.5% (w/v) was added to make soymilk. Soymilk was heated at 85°C for 15 min for sterilization and then cooled to 37°C. Subsequently, the soymilk was inoculated with 10% (w/v) *L. amylolyticus* L6 and incubated at 37°C for 24 hr. The growth curve was plotted according to the viable counts determined at 0 hr, 2 hr, 4 hr, 6 hr, 8 hr, 10 hr, 12 hr, 14 hr, and 16 hr during fermentation (Tang et al., 2007). All analyses were performed in triplicate.

### 2.3 Transcriptomic analysis

The fermented soymilk was sampled at the fermentation time of 4 hr, 7 hr, and 10 hr corresponding to the lag phase, logarithmic phase, and stationary phase, respectively. Three parallel samples were obtained in each sampling point. The quality and integrity of total RNA were assessed by 1% agarose gels and RNA 6000 Nano Assay Kit of the Bioanalyzer 2100 system. Probes were used to purify mRNA from the total RNA of prokaryotic samples. Fragmentation was carried out using divalent cations under hyperthermal temperature in first strand synthesis reaction buffer (5X). Synthesis of first strand complementary DNA (cDNA) was performed with random hexamer primer and Moloney murine leukemia virus (M‐MuLV) reverse transcriptase. The second strand was synthesized by DNA Polymerase I and M‐MuLV reverse transcriptase. The 3’ ends of DNA fragments were adenylated and then ligated to the adaptor with hairpin loop structure for hybridization. The cDNA library fragments with 350–400 bp were selected and purified with AMPure XP system. Polymerase chain reaction (PCR) was carried out with Phusion High‐Fidelity DNA polymerase and the PCR products were purified with AMPure XP system. Finally, library quality was evaluated with Agilent 2100 Bioanalyzer system (Cheng et al., 2019). Gene descriptions and annotations were performed in the Genome Database of *L*. *amylolyticus* strain L6 in National Center for Biotechnology Information (NCBI) (https://www.ncbi.nlm.nih.gov) with GenBank Accession Number of CP020457.1. The annotated genes were then used to predict biochemical pathways. Kyoto Encyclopedia of Genes and Genomes (KEGG) pathways and gene ontology (GO) terms were retrieved from the KEGG database (http://www.kegg.jp/kegg) and gene ontology (GO) database (http://geneontology.org), respectively.

Real‐time quantitative PCR (RT–qPCR) was applied to verify the accuracy of transcriptomic results. Primers were designed and synthesized according to gene sequences on NCBI (Table S1). The gene‐expression levels were calculated via the 2^—△△Ct^ method (Xu et al., 2015), which was used to compare with the sequencing results of the transcriptome.

### 2.4 Sugars and organic acid

The determination of sugars, including sucrose, glucose, fructose, galatose, raffinose, and stachyose in fermented soymilk, was performed by high‐performance liquid chromatography (HPLC) according to the National Standard of China GB/T 22,221–2008. The samples with a volume of 5 ml were collected at 0 hr, 4 hr (lag phase), 7 hr (log phase), and 10 hr (stationary phase) and then centrifuged at 10,000 r/min for 10 min. The supernatant was filtered through a 0.22‐µm syringe membrane into HPLC vials for testing. HPLC was carried out on Thermo‐Ultimate 3000 equipped with HP‐NH2 column (4.6 mm×250 mm, 5 μm) and differential refraction detector RefractoMax 520. Acetonitrile (68%)–deionized water (32%) were used as mobile phase with a flow rate of 1.0 ml/min. The detection wavelength was 280 nm, and the column temperature was set at 35°C. The standard of sucrose, glucose, fructose, galactose, raffinose, and stachyose (Sigma Chemical Co., Ltd, Guangzhou, China) was dissolved in deionized water and transferred to a 10‐mL volumetric flask for gradient dilution. The equation parameters of standard curve were used to determine the concentration of sugar in fermented soymilk.

The acidity values of fermented soymilk samples were determined according to a previous description (Fei, Liu, et al., 2017). The distilled water with a volume of 20 ml was added to 10‐mL collected samples. Then, 30 ml of mixture was mixed with 0.5 ml of phenolphthalein indicator to titrate the amount of acidity against NaOH solution (0.1 mol/L). All analyses were performed in triplicate.

The content of lactic acid and acetic acid in fermented soymilk was detected by HPLC according to GB/T 5009.157–2003. The samples with a volume of 5 ml were collected at 0 hr, 4 hr (lag phase), 7 hr (log phase), and 10 hr (stationary phase) and then centrifuged at 10,000 r/min for 10 min. The supernatant was filtered through a 0.22‐µm syringe membrane into HPLC vials for testing. HPLC was performed on Agilent 1100 equipped with Luna C18(2) 100A column (4.6 mm×250 mm, 5 μm) and VWD 3100 ultraviolet detector. KH_2_PO_4_ (95%) with a concentration of 10 mmol/L‐methanol (5%) was used as mobile phase with a flow rate of 0.5 ml/min. The detection wavelength was 210 nm, and the column temperature was set at 25°C. The standard of lactic acid and acetic acid (Sigma Chemical Co., Ltd, Guangzhou, China) was dissolved in deionized water and transferred to a 10‐mL volumetric flask for gradient dilution. The equation parameters of standard curve were used to determine the concentration of organic acid in fermented soymilk.

### 2.5 Analysis of isoflavones and amino acids by HPLC

The content of isoflavones in fermented soymilk was determined by HPLC according to our previous report (Fei et al., 2018; Fei, Liu, et al., 2017). The samples were collected at 0 hr, 4 hr (lag phase), 7 hr (log phase), and 10 hr (stationary phase) and then centrifuged at 10,000 r/min for 10 min at 4 ℃. The supernatant (4 ml) was transferred to a 10‐mL volumetric flask, diluted with methanol to the constant volume, and extracted with sonication for 1 hr. The resulting extracts were filtered through a 0.22‐µm membrane into HPLC vials for HPLC testing.

The content of amino acid in fermented soymilk was determined by HPLC according to Agilent AdvanceBio AAA method (www.agilent.com/chem/advancebioaaa). Briefly, the pretreated samples were derivatized with o‐phthalaldehyde (OPA), and the specific operations were carried out according to the method provided by Agilent. Analysis of amino acid by HPLC was carried out on Agilent 1100 equipped with an Agilent AdvanceBio AAA amino acid column (4.6 mm×100 mm, 2.7 μm) under isocratic elution. Na_2_HPO_4_ with a concentration of 0.01 mol/L and acetonitrile–methanol solution (acetonitrile:methanol:water 45:45:10) were used as mobile phase A and mobile phase B, respectively, with a flow rate of 1.5 ml/min. The detection wavelength was 338 nm, and the column temperature was set at 40°C. The standard of amino acids (Sigma Chemical Co., Ltd, Guangzhou, China) was dissolved in deionized water and transferred to a 10‐mL volumetric flask for gradient dilution. The equation parameters of standard curve were used to determine the concentration of amino acids in fermented soymilk.

### 2.6 Statistical analysis

Analyses were performed using SPSS (SPSS Inc., Chicago, IL, USA, V23.0.0). One‐way analysis of variance (ANOVA) was performed using to compare between groups, which was considered statistically significant at the *p* <.05 level.

## 3 RESULTS AND DISCUSSION

### 3.1 Growth characteristics of *L. amylolyticus* L6 in soymilk

**FIGURE 1:**
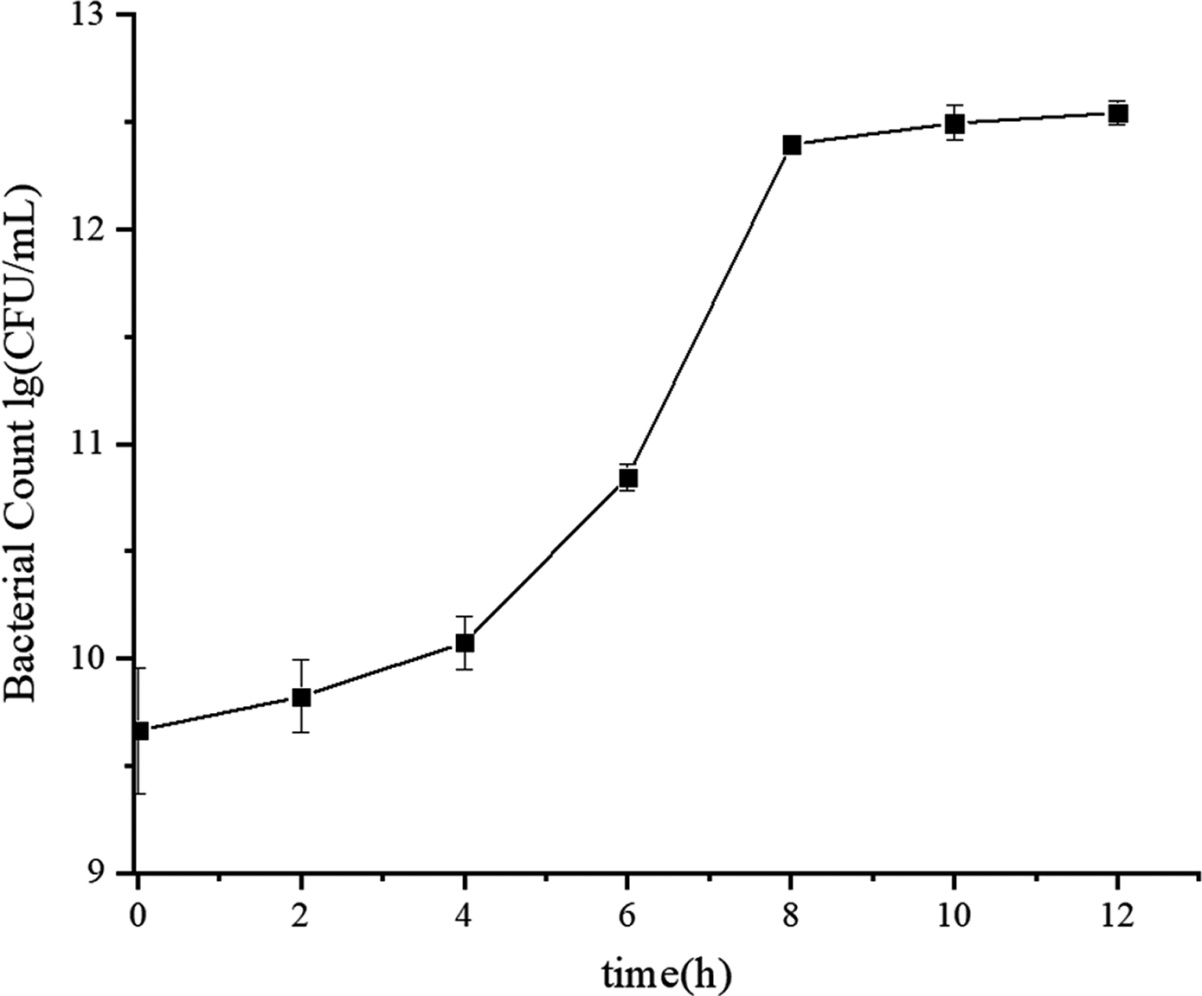
Growth of *L.amylolyticus* L6 in soymilk incubated at 37°C

The growth curve of *L. amylolyticus* L6 in soymilk was plotted according to viable counts (Figure 1). *L. amylolyticus* L6 started to grow after 2 hr inoculation in soymilk. It needed approximately 4 hr for bacteria to grow from lag phase into the logarithmic phase. Bacteria grew into the stationary phase at the time of 10 hr with a cell concentration of 10^12^ CFU/mL. It was reported that *Lactobacillus casei* Zhang grew from the lag phase into the logarithmic phase at a time of 3 hr and reached stationary phase at 14 hr with a cell concentration of 10^9^ CFU/mL (Wang et al., 2012). *L. amylolyticus* L6 need less time than *L*. *casei* Zhang to grow into stationary phase, while L6 could produce more bacterial cells in soymilk than *L*. *casei* Zhang. That might be because tofu‐whey isolated *L. amylolyticus* L6 is more adaptable in the soymilk‐based ecosystem than koumiss‐isolated *L*. *casei* Zhang (Fei, Liu, et al., 2017).

### 3.2 Gene‐expression profiles of *L. amylolyticu*s L6 during fermentation in soymilk

**FIGURE 2:**
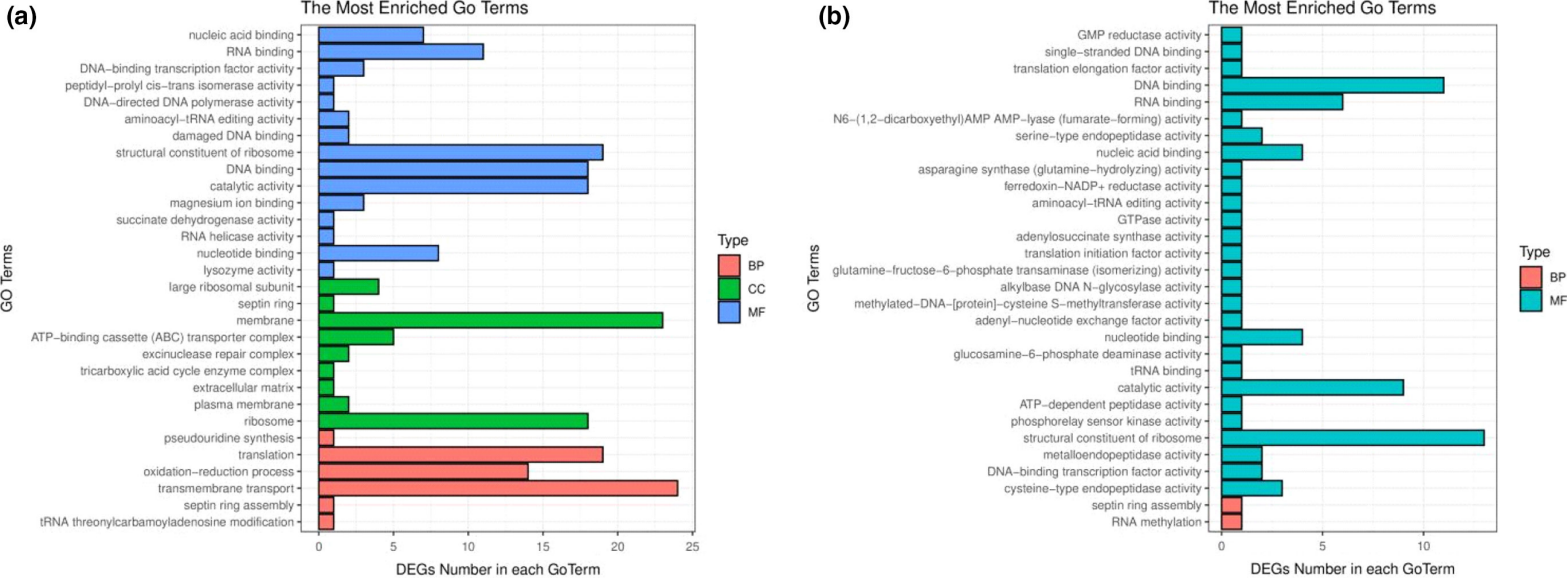
Significantly differentially expressed genes (SDGEs) between different growth phase based on gene ontology (GO) analysis. Logarithmic phase versus Lag phase (a), Stable phase versus Logarithmic phase(b); BP, CC, and FF refer to biological process, cellular component, and molecular function, respectively

**FIGURE 3:**
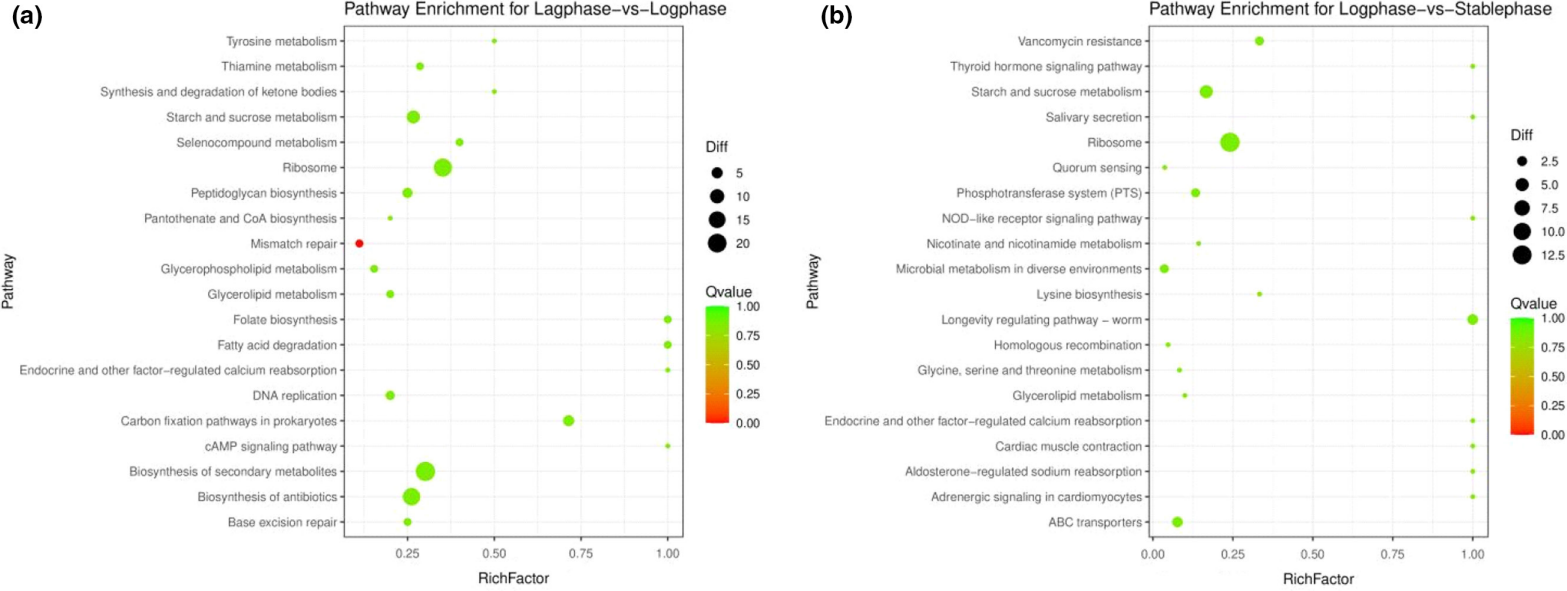
Scatter plot of Kyoto Encyclopedia of Genes and Genomes (KEGG) pathway enrichment analysis for different growth phases. Rich factor is the ratio of the number of differentially expressed genes (DEGs) annotated to the Pathway Term to the number of genes annotated to the Pathway entry

The gene‐expression profiles of *L.amylolyticus* L6 during growth in the soymilk ecosystem were investigated by the RNA‐sequencing (RNA‐seq). Our research mainly focused on the comparative transcriptomic analysis of different growth phases next to each other. A total of 313 significantly differentially expressed genes (SDEGs) were identified. There were 260 SDEGs in logarithmic phase versus lag phase and 171 SDEGs identified in the stationary phase versus logarithmic phase (FigureS 1). The SDEGs of *L.amylolyticus* L6 in logarithmic phase versus lag phase were functionally categorized, indicating that SDEGs mainly enriched in biological process (transmembrane transport, oxidation and reduction, and translation), cellular component (ribosome and membrane), and molecular function (structural constituent of ribosome, nucleotide binding, and catalytic activity) (Figure 2a). In the stationary phase compared to the logarithmic phase, SDEGs mainly enriched in molecular function, such as structural constituent of ribosome, nucleotide binding, and catalytic activity (Figure 2b). The pathway enrichment for different growth phases is shown in Figure 3 according to the KEGG pathway database. As for logarithmic phase versus Lag phase group, the pathway of SDEGs enriched in starch and sucrose metabolism, fatty acid degradation, ribosome, biosynthesis of secondary metabolites, and folate biosynthesis. The pathway of SDEGs in stationary versus logarithmic phase group enriched in ribosome, starch, and sucrose metabolism, adenosine triphosphate (ATP)‐binding cassette (ABC) transporter, phosphotransferase system (PTS), and amino acid metabolism.

**FIGURE 4:**
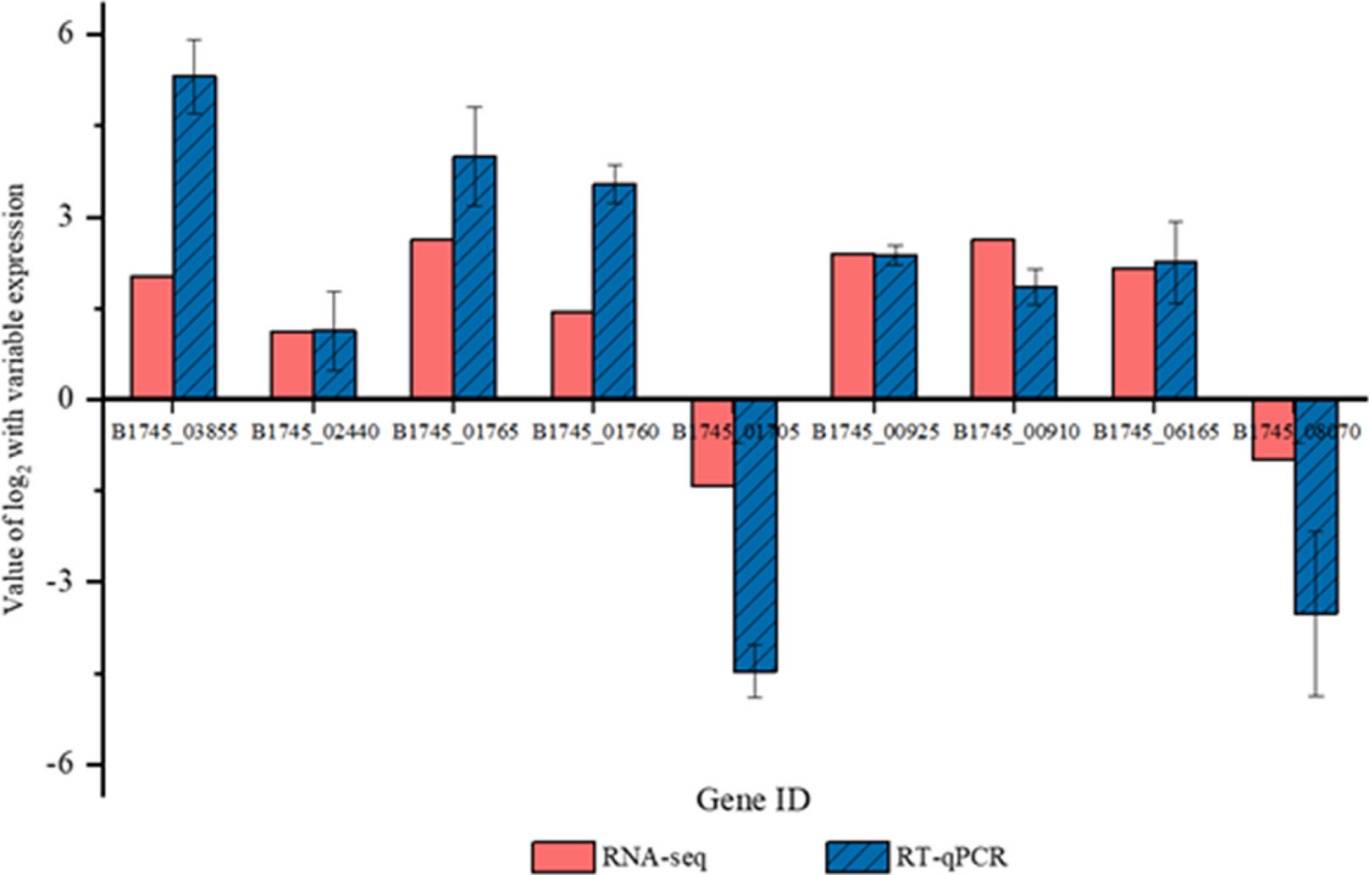
Reliability analysis between RNA‐sequencing (RNA‐seq) and real‐time quantitative polymerase chain reaction (RT‐qPCR)

In order to determine the reliability of transcriptomic results, expression changes of nine target genes (B1704_03855, B1704_02440, B1704_01765, B1704_01760, B1704_00925, B1704_00910, and B1704_06165) between lag phase and stationary phase were selected for detection. The result indicated a high consistency between platform of RNA‐seq and real‐time quantitative polymerase chain reaction (RT–qPCR), proving the validity of RNA‐seq data (Figure 4).

### 3.3 Carbon metabolism of *L. amylolyticus* L6

**FIGURE 5:**
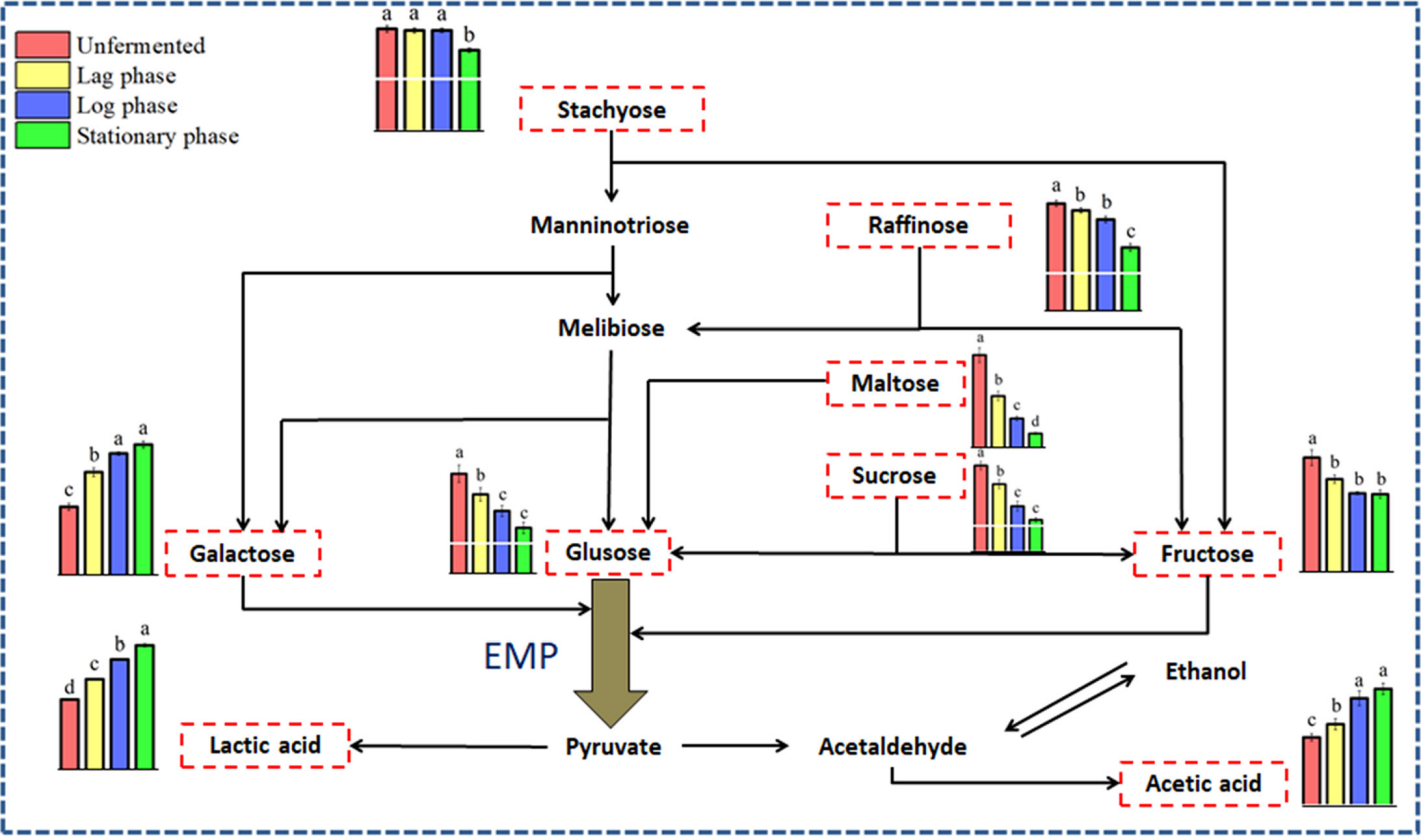
Schematic representation of the presumptive carbohydrate metabolic pathways in *L. amylolyticus* L6 during fermentation in soymilk. Changes in the amounts of the substances (average of three replicates) are represented by histograms. Different colored bars represent period: unfermented (red), lag phase (yellow), log phase (blue), and stationary phase (green)

It has been reported that *Lactobacillus amylolyticus* could metabolize various carbohydrates such as dextrin, fructose, galactose, glucose, maltose, mannose, sucrose, melibiose, and raffinose, and in some cases salicine, esculin, amygdalin, and starch (Bohak et al., 1998; Fei et al., 2018). Soymilk mainly contained four different kinds of sugars, including glucose, sucrose, raffinose, and stachyose (Table 1). To provide a guide for industrial applications of *L. amylolyticus* L6 in fermenting soymilk, 1.5% (w/v) of glucose was added to provide enough carbon source for the growth of L6. The metabolism of carbohydrate to produce organic acid by *L.amylolyticus* L6 during its fermentation in soymilk is shown in Figure 5. The results indicated that four kinds of sugars reduced significantly (*p* <.5) during the fermentation and the main carbon sources used for the growth of *L.amylolyticus* L6 were sucrose and glucose (Table 1 and Figure 5). Many genes related to glucose metabolism were significantly up‐regulated in logarithmic phase, such as genes coding for PTS *β*‐glucoside transporter (B1745_01765), 6‐phospho‐alpha‐glucosidase (B1745_05130), gluconate kinase (B1745_01565), and glucose‐6‐phosphate dehydrogenase (B1745_01805) (Table 2). However, several genes involved in sucrose transportation, especially sugar ABC transporters (B1745_06760, B1745_06745 and B1745_06745), and galactose metabolism (B1745_05485 and B1745_05490) were significantly down‐regulated in logarithmic phase. Microbes intend to utilize easily metabolizable carbohydrate and inhibit the metabolism of the other carbohydrate by down‐regulating the expression of related genes (Luesink et al., 1998), the phenomenon of which, called carbon catabolite repression (CCR), has been widely found in lactic acid bacteria (LAB) (Görke & Stülke, 2008; Wang et al., 2012). In the stationary phase, few genes related to glucose metabolism were induced while many genes involved in sucrose (B1745_04485, B1745_04615, B1745_06775), raffinose, and stachyose utilization were found to be significantly up‐regulated (Table 3). Among these sugars, only the content of galactose increased slightly (Table 1), which was due to the partial hydrolysis of raffinose and stachyose by α‐galactosidase (B1745_RS08070) (Table 2). This phenomenon has also been reported in several researches of soybean products fermented by LAB (Battistini et al., 2018; Elghali et al., 2014; Xia et al., 2019). The production of energy for *L*. *amylolyticus* L6 is mainly through the Embden–Meyerhof–Parnas pathway (EMP). The gene (B1745_01805) of glucose 6‐phosphate dehydrogenase that is the key regulatory enzyme of the Hexose Monophosphate Pathway (HMP) was highly expressed in the log phase, indicating that HMP was also indispensable in the glycometabolism of *L*. *amylolyticus* L6. Besides, two genes (B1745_05365 and B1745_06945) relevant to ATP production were also significantly up‐regulated.

**TABLE 1:** Changes of sugar and organic acid in soymilk fermented with *L*. *amylolyticus* L6

		Unfermented	Lag phase	Logarithmic phase	Stationary phase
Sugars (g/L)	Sucrose	3.75 ± 0.09^a^	3.36 ± 0.09^b^	2.89 ± 0.10^c^	2.61 ± 0.05^c^
	Glucose	8.01 ± 0.15^a^	7.65 ± 0.12^ab^	7.36 ± 0.10b^c^	7.06 ± 0.14^c^
	Fructose	0.13 ± 0.01^a^	0.11 ± 0.00^b^	0.09 ± 0.00^b^	0.09 ± 0.00^b^
	Raffinose	0.59 ± 0.00^a^	0.59 ± 0.01^ab^	0.57 ± 0.01^b^	0.53 ± 0.05^c^
	Stachyose	2.89 ± 0.02^a^	2.88 ± 0.02^a^	2.88 ± 0.01^a^	2.76 ± 0.01^b^
	Galactose	0.23 ± 0.01^c^	0.35 ± 0.01^b^	0.41 ± 0.01^a^	0.44 ± 0.02^a^
Organic acids (g/L)	Lactic acid	2.62 ± 0.04^d^	3.38 ± 0.04^c^	4.12 ± 0.04^b^	4.65 ± 0.06^a^
	Acetic acid	0.36 ± 0.02^c^	0.43 ± 0.03^b^	0.57 ± 0.04^a^	0.62 ± 0.03^a^
Acidity (°)		45.33 ± 1.14^c^	47.64 ± 1.86^c^	88.42 ± 2.31^b^	95.88 ± 2.65^a^
pH		6.7 ± 0.1^a^	6.6 ± 0.1^a^	5.5 ± 0.2^b^	4.3 ± 0.2^c^

Note

Data are the mean ±standard deviation (*n* = 3). Means in the same column with different superscript letters (a–d) are significantly different (*p* <.05)

**TABLE 2:** Genes differentially expressed in the logarithmic phase compared to lag phase

Function group and ORF	Gene	Description	Value of log_2_ variable expression
Genes up‐regulated			
Carbohydrate transport and metabolism			
B1745_05615	*nagZ*	beta‐*N*‐acetylhexosaminidase	3.54
B1745_05130	*glvA*	6‐phospho‐alpha‐glucosidase	3.26
B1745_03165	*ldh*	L‐lactate dehydrogenase	3.18
B1745_05695	*adhE*	acetaldehyde dehydrogenase /alcohol dehydrogenase	2.67
B1745_RS08070	*galA*	alpha‐galactosidase	2.32
B1745_01765	*scrA*	PTS beta‐glucoside transporter subunit EIIBCA	1.98
B1745_04615	*spp*	HAD family hydrolase	1.93
B1745_03820	*gyaR*	hypothetical protein	1.80
B1745_03825	*spp*	sugar‐phosphatase	1.69
B1745_01805	*zwf*	glucose−6‐phosphate dehydrogenase	1.62
B1745_06170	*gpmB*	histidine phosphatase family protein	1.58
B1745_07215	*‐*	transcriptional regulator	1.44
B1745_02045	*dacC*	D‐alanyl‐D‐alanine carboxypeptidase	1.44
B1745_02005	*bcrC*	phospholipid phosphatase	1.44
B1745_03140	*fumC*	class II fumarate hydratase	1.43
B1745_04590	*murB*	UDP‐*N*‐acetylenolpyruvoylglucosamine reductase	1.38
B1745_04460	*glgC*	YebC/PmpR family DNA‐binding transcriptional regulator	1.36
B1745_04525	*spp*	HAD family hydrolase	1.33
B1745_03145	*frdA*	flavocytochrome c	1.32
B1745_02465	*galE*	UDP‐glucose−4‐epimerase	1.30
B1745_07245	*poxL*	pyruvate oxidase	1.29
B1745_04860	*pgl*	3‐carboxymuconate cyclase	1.29
B1745_06310	*rpe*	ribulose phosphate epimerase	1.22
B1745_00870	*‐*	aldose 1‐epimerase	1.19
B1745_07130	*bdhAB*	aldo/keto reductase	1.19
B1745_03235	*lysozyme*	lysin	1.12
B1745_01565	*gntK, idnK*	gluconate kinase	1.09
B1745_04605	*pta*	phosphate acetyltransferase	1.09
B1745_05160	*rpiA*	ribose 5‐phosphate isomerase A	1.03
B1745_04420	*ackA*	acetate kinase	1.00
B1745_03855	*bglA*	6‐phospho‐beta‐glucosidase	1.00
B1745_05365	*zntA*	copper‐translocating P‐type ATPase	1.09
B1745_06945	*‐‐*	cadmium‐translocating P‐type ATPase	2.16
Amino acids transport and metabolism			
B1745_06855	*asnA*	aspartate‐‐ammonia ligase, asparagine biosynthetic process	4.16
B1745_02860	*pepO*	peptidase M13	2.44
B1745_00965	*hsp20*	heat‐shock protein Hsp20	2.39
B1745_04695	*clpP*	ATP‐dependent Clp protease proteolytic subunit	2.35
B1745_04960	*clpE*	Clp protease ClpE	2.25
B1745_05185	*glnP*	glutamine ABC transporter permease	2.16
B1745_03105	*att*	amino acid permease	2.14
B1745_00925	*oppB*	peptide ABC transporter substrate‐binding protein	2.07
B1745_00550	*htpX*	zinc metalloprotease HtpX	1.97
B1745_06070	*pepT*	peptidase T	1.89
B1745_00950	*oppF*	ABC transporter ATP‐binding protein	1.83
B1745_03200	*prmA*	ribosomal protein L11 methyltransferase	1.81
B1745_00910	*pepC*	aminopeptidase	1.67
B1745_01265	*clpC*	ATP‐dependent Clp protease ATP‐binding protein ClpC	1.63
B1745_04890	*‐*	transcriptional activator, Rgg/GadR/MutR family domain‐containing protein	1.60
B1745_05190	*glnM*	glutamine ABC transporter permease	1.60
B1745_02545	*pepX*	dipeptidyl‐peptidase	1.56
B1745_02045	*dacD*	D‐alanyl‐D‐alanine carboxypeptidase	1.44
B1745_06875	*‐*	amino acid permease	1.44
B1745_00945	*oppD*	peptide ABC transporter ATP‐binding protein	1.43
B1745_RS07280	*lysC*	aspartate kinase	1.41
B1745_06870	*att*	amino acid permease	1.40
B1745_04855	*atpA*	haloacid dehalogenase	1.40
B1745_00955	*pepC*	aminopeptidase	1.38
B1745_05195	*glnH*	glutamine ABC transporter substrate‐binding protein	1.32
B1745_01775	*groEL*	chaperonin GroEL	1.23
B1745_06165	*‐*	metalloprotease	1.18
B1745_05200	*GlnP*	glutamine ABC transporter permease	1.08
B1745_00920	*oppA*	peptide ABC transporter substrate‐binding protein	1.07
B1745_07210	*cth*	aluminum resistance protein	1.05
B1745_04725	*uvrB*	excinuclease ABC subunit B	1.02
B1745_00940	*oppC*	peptide ABC transporter permease	1.01
B1745_00320	*gadC*	glutamate:gamma‐aminobutyrate antiporter	1.79
Lipid metabolism, inorganic ion transport and stress response			
B1745_05970	*‐*	esterase	1.41
B1745_00245	*‐*	esterase	1.03
B1745_02830	*‐*	acyl‐CoA thioesterase	1.40
B1745_05670	*‐*	biotin carboxylase	1.58
B1745_00100	*mgtC*	putative Mg^2+^ transporter family protein	1.26
B1745_00845	*pot*	potassium transporter	1.36
B1745_05305	*amt*	ammonium transporter	1.97
B1745_06945	‐	cadmium‐translocating P‐type ATPase	2.16
Genes down‐regulated			
Carbohydrate transport and metabolism			
B1745_06195	*‐*	hypothetical protein	−1.03
B1745_05125	*glvB*	PTS alpha‐glucoside transporter subunit IICB	−1.06
B1745_05025	*atoB*	3‐ketoacyl‐CoA thiolase	−1.08
B1745_00695	*ddl*	D‐alanine–D‐alanine ligase A	−1.27
B1745_05485 B1745_05490	*tagA*	galactosyltransferase	−1.33 −1.56
B1745_06760	*Malk*	sugar ABC transporter ATP‐binding protein	−1.36
B1745_02355	*acyP*	acylphosphatase	−1.50
B1745_06765	*pgmB*	beta‐phosphoglucomutase	−1.61
B1745_06730	*gpmB*	hypothetical protein	−2.38
B1745_06745	*ganQ*	sugar ABC transporter permease	−2.58
B1745_06750	*ganP*	sugar ABC transporter permease	−2.79
Amino acids transport and metabolism			
B1745_01435	*rpoA*	DNA‐directed RNA polymerase subunit alpha	−1.02
B1745_02350	*yidC*	insertase	−1.07
B1745_04680	*‐*	amino acid permease	−1.14
B1745_05775	*secG*	preprotein translocase subunit SecG	−1.17
B1745_06880	*lepB*	S26 family signal peptidase	−1.19
B1745_04160	*valS*	valine‐tRNA ligase	−1.22
B1745_01405	*secY*	preprotein translocase subunit SecY	−1.38
B1745_02435	*cth*	aluminum resistance protein	−1.74
B1745_03815	*‐*	amino acid permease	−2.08

**TABLE 3:** Genes differentially expressed in the stationary phase compared to logarithmic phase

Function group and ORF	Gene	Description	Expression ratio
Genes up‐regulated			
Carbohydrate transport and metabolism			
B1745_04550	*glmS*	glutamine‐‐fructose−6‐phosphate transaminase (isomerizing)	2.34
B1745_04615	*spp*	HAD family hydrolase	1.91
B1745_04860	*pgl*	3‐carboxymuconate cyclase	1.30
B1745_06170	*gpmB*	histidine phosphatase family protein	1.26
B1745_04485	*spp*	sugar‐phosphatase	1.20
B1745_06775	*nplT*	alpha‐glycosidase	1.14
Amino acid transport and metabolism			
B1745_04550	*glmS*	glutamine‐fructose−6‐phosphate transaminase (isomerizing)	2.34
B1745_00615	*adaB*	cysteine methyltransferase	2.00
B1745_01515	*pepC*	aminopeptidase	1.55
B1745_06860	*‐*	amino acid permease	1.54
B1745_00965	*hsp20*	heat‐shock protein Hsp20	1.52
B1745_03805	*uvrC*	excinuclease ABC subunit C	1.45
B1745_06885	*clpE*	ATP‐dependent Clp protease ATP‐binding subunit	1.40
B1745_04960	*clpE*	Clp protease ClpE	1.33
B1745_04860	*pgl*	3‐carboxymuconate cyclase	1.30
B1745_01540	*cysS*	cysteine‐tRNA ligase	1.29
B1745_04890	*‐*	transcriptional activator, Rgg/GadR/MutR family domain‐containing protein	1.23
B1745_03010	*grpE*	nucleotide exchange factor GrpE	1.23
B1745_00550	*htpX*	zinc metalloprotease HtpX	1.19
B1745_04855	*atpA*	haloacid dehalogenase	1.18
B1745_01260	*‐*	histidine kinase	1.16
B1745_00985	*‐*	CPBP family intramembrane metalloprotease	1.15
B1745_04695	*clpP*	ATP‐dependent Clp protease proteolytic subunit	1.14
B1745_07110	*pcp*	pyroglutamyl‐peptidase I	1.06
B1745_06165	*‐*	metalloprotease	1.05
B1745_00910	*pepC*	aminopeptidase	1.03
Lipid metabolism, inorganic ion transport, and stress response			
B1745_02830	*‐*	acyl‐CoA thioesterase	2.30
B1745_01775	*groEL*	chaperone	1.22
B1745_03010	*grpE*	nucleotide exchange factor	1.23
B1745_03015	*dnaK*	molecular chaperone	1.10
B1745_00745	*uspA*	universal stress protein	1.14
B1745_01850	*trxA*	thioredoxin	1.20
Genes down‐regulated			
Carbohydrate transport and metabolism			
B1745_01165	*murF*	UDP‐*N*‐acetylmuramoyl‐tripeptide‐‐D‐alanyl‐D‐ alanine ligase	−1.02
B1745_05730	*manY*	PTS mannose/fructose/sorbose transporter subunit IIC	−1.04
B1745_05730	*manY*	PTS mannose/fructose/sorbose transporter subunit IIC	−1.04
B1745_05735	*manX*	PTS mannose transporter subunit IIAB	−1.15
B1745_05130	*glvA*	6‐phospho‐alpha‐glucosidase	−1.33
B1745_07135	*nagB*	glucosamine−6‐phosphate deaminase	−2.70
Amino acid transport and metabolism			
B1745_04680	*‐*	amino acid permease	−1.01
B1745_05320	*livB*	branched‐chain amino acid transport system II carrier protein	−1.02
B1745_05735	*manX*	PTS mannose transporter subunit IIAB	−1.15
B1745_02915	*tsf*	translation elongation factor Ts	−1.17
B1745_03935	*‐*	peptide‐binding protein	−1.24
B1745_02235	*thrS*	threonine‐tRNA ligase	−1.41
B1745_00785	*asnB*	asparagine synthase (glutamine‐hydrolyzing)	−1.47
B1745_00270	*brnQ*	branched‐chain amino acid ABC transporter permease	−1.69
Lipid metabolism, inorganic ion transport, and stress response			
B1745_00715	‐	acetylesterase	−1.10

During the fermentation, the acidity of soymilk increased significantly from 45.33° to 95.88° (Table 1). The acidity increment was mainly derived from lactic acid with its content increased from 2.62g/L to 4.65g/L (stationary phase) (*p* <.05). In addition, the content of acetic acid also increased slightly. Organic acid production was produced by *L. amylolyticus* L6 through the consumption of sugars in the soymilk (Wang et al., 2012; Xia et al., 2019). The production of acetic acid indicated that this strain was a facultatively heterofermentative bacterium. After glucose is converted into pyruvate by glycolysis, it can generate lactic acid through the action of lactate dehydrogenase under anaerobic conditions (Figure 5). RNA‐Seq results showed that the expression of *LDH* gene (B1745_03165) encoding L‐lactate dehydrogenase was significantly increased in the log phase (Table 2). In addition, it was also observed that *adhE* (B1745_05695) encoding acetaldehyde hydrogenase related to acetic acid production was up‐regulated in the log phase. Therefore, high expression of *LDH* and *adhE* genes promotes the production of lactic acid and acetic acid which are important for coagulating soymilk.

### 3.4 Nitrogen metabolism and biosynthesis

Due to the lack of various biosynthetic pathways, especially amino acid synthesis pathways, LAB generally need various nutritional ingredients and therefore they are usually found in nutrient‐rich environments, such as vegetables, meat, and milk (Fernández & Zúñiga, 2006). Amino acids as an important nitrogen resource for LAB played important roles in physiological functions such as intracellular pH maintenance, stress resistance, and energy generation (Lei et al., 2018; Slonczewski et al., 2009). As a consequence, the proteolytic enzyme system serves a key role for LAB to grow in protein‐rich soymilk. A total of 17 kinds of amino acids were detected in the fermented soymilk, including seven kinds of essential amino acids (EAAs) and 10 kinds of nonessential amino acids (NEAAs) (Table 4). The content of EAAs decreased gradually along with the fermentation and reached 177.26 mg/L in the stationary phase. But the content of total amino acids and NEAAs increased significantly and reached the highest 668.38 mg/L and 187.40 mg/L in the logarithmic phase respectively. Although the content of total amino acids and NEAAs decreased slightly in the stationary phase, it was still higher than that of unfermented soymilk. The increase of free amino acid content in the soymilk fermented by different lactobacilli and their mixes has been widely reported (Ceh et al., 2020; Song et al., 2008). Besides, the content of glutamate and arginine was higher than those of other amino acids in unfermented and fermented soymilk, accounting for approximately 40% content of total amino acid. This phenomenon has been found in soy powder yoghurt fermented by *L. brevis* WCP02 and *L. plantarum* P120 that content of arginine is the highest (reached 380 mg/g), and accounted for almost 50% content of the total amino acid in soy powder yoghurt (Ceh et al., 2020). Therefore, fermentation of soymilk by *L. amylolyticus* L6 could promote the hydrolysis of protein into amino acid, improving the nutritional quality and digestibility of soymilk.

**TABLE 4:** Changes of amino acids in soymilk fermented with *L*. *amylolyticus* L6

Free amino acids (mg/L)	Period			
	Unfermented	Lag phase	Log phase	Stationary phase
Essential amino acids				
Lysine	39.68 ± 1.72^a^	36.60 ± 1.21^b^	35.13 ± 0.28^bc^	33.06 ± 0.99^c^
Phenylalanine	29.62 ± 0.79^ab^	30.39 ± 0.86^a^	28.87 ± 2.03^ab^	26.79 ± 1.39^b^
Methionine	47.04 ± 1.50^a^	43.05 ± 1.79^b^	43.72 ± 1.09^ab^	42.30 ± 1.85^b^
Threonine	8.59 ± 0.21^c^	9.31 ± 0.25^ab^	9.59 ± 0.25^a^	9.01 ± 0.22^bc^
Isoleucine	20.32 ± 1.33	21.28 ± 0.92	20.80 ± 0.53	19.57 ± 2.50
Leucine	50.91 ± 1.98^a^	48.33 ± 1.76^ab^	46.65 ± 1.20^b^	43.40 ± 2.11^bc^
Valine	5.74 ± 0.33^a^	2.19 ± 0.22^d^	2.63 ± 0.08^c^	3.13 ± 0.08^b^
Total of EAA	201.90 ± 7.95^a^	191.14 ± 8.18^b^	187.40 ± 4.82^b^	177.26 ± 9.56^c^
Nonessential amino acids				
Asparagine	21.70 ± 0.76^a^	14.10 ± 1.14^b^	10.64 ± 1.02^c^	5.48 ± 0.52^d^
Glutamate	102.04 ± 3.21^b^	133.14 ± 2.90^a^	133.30 ± 3.15^a^	129.07 ± 0.99^a^
Serine	23.27 ± 0.82^b^	26.16 ± 1.63^a^	27.98 ± 0.92^a^	27.53 ± 0.66^a^
Histidine	4.65 ± 0.41^c^	7.03 ± 0.23^a^	6.82 ± 0.13^a^	6.15 ± 0.25^b^
Glycine	48.10 ± 1.61^d^	61.88 ± 1.93^c^	76.37 ± 1.00^b^	83.97 ± 1.12^a^
Arginine	125.25 ± 2.85^a^	121.10 ± 2.56^a^	120.45 ± 2.65^ab^	115.86 ± 1.73^b^
Alanine	60.34 ± 1.87^a^	51.74 ± 2.00^b^	53.91 ± 1.00^b^	52.86 ± 0.98^b^
Tyrosine	11.42 ± 0.19^d^	16.97 ± 0.24^a^	16.23 ± 0.51^b^	15.09 ± 0.41^c^
Cysteine	0.79 ± 0.06^d^	1.21 ± 0.04^c^	1.92 ± 0.09^b^	3.18 ± 0.05^a^
Proline	32.66 ± 1.55	32.83 ± 1.84	33.35 ± 0.67	32.69 ± 1.05
Total of NEAAs	430.23 ± 13.34^c^	466.16 ± 14.53^b^	480.99 ± 11.14^a^	471.86 ± 7.77^ab^
Total amino acids	632.12 ± 21.28^c^	657.30 ± 22.70^ab^	668.38 ± 15.97^a^	649.12 ± 17.33^bc^

Note

Data are the mean ±standard deviation (*n* = 3). Means in the same row with different superscript letters (a–d) are significantly different (*p* <.05)

The proteolytic system of LAB generally consisted of protease, transport systems of amino acid or peptides, and peptidases (Wang et al., 2012). The protein in soymilk was first hydrolyzed by protease into amino acids and peptides, which were then transported to cytoplasm by transport systems. Finally, the translocated peptides were degraded by peptidases (Savijoki et al., 2006). Transcriptomic results indicated that the expression of *clpP* (B1745_04695), *clpC* (B1745_01265), and *clpE* (B1745_04960) genes encoding subunits of caseinolytic protease (*Clp*) previously identified in *Lactobacillus plantarum* IIA‐1AS (Mega et al., 2020) was highly induced in the logarithmic and stationary phase. Meanwhile, two genes (B1745_00550 and B1745_06165) coding for metalloprotease were also found to be up‐regulated in the logarithmic and stationary phase. Besides, the gene of another intramembrane metalloprotease showed higher induction levels in the stationary phase than in the logarithmic phase. These highly expressed protease genes indicated the strong proteolytic ability of *L. amylolyticus* L6 in the fermentation of soymilk.

The transport of peptides into the cell is an essential step for LAB multiplying in soymilk (Hagting, 1995). Transcriptomic data showed that genes involved in the transport and hydrolysis of peptide in the cytoplasm also exhibited high expression levels (Tables 2 and 3). The gene cluster *oppDFBCA* encoding the oligopeptide transport system (Opp) and *PepC* encoding the aminopeptidase, which have been identified in an operon of *Lactococcus lactis* (Tynkkynen et al., 1993), were found to be up‐regulated in soymilk‐grown *L*. *amylolyticus* L6 cells. Five genes in *Opp* operon were highly expressed, including B1745_00920 (*OppA* coding for substrate‐binding proteins), B1745_00925 and B1745_00940 (*OppB* and *OppC* coding for membrane proteins), and B1745_00945 (*OppD* and *OppF* coding for ATP‐binding proteins). Meanwhile, *pepC* coding for aminopeptidase that could hydrolyze oligopeptide into amino acid also exhibited high expression levels. Additionally, another five peptidase genes were highly induced in the logarithmic phase, which include two peptidases (*pepO*, B1745_02860; *pepT*, B1745_06070), two aminopeptidases (*pepC*, B1745_00910 and B1745_00955), and a dipeptidyl‐peptidase (*pepX*, B1745_02545). Dipeptidyl‐peptidase (*pepX*, B1745_02545) identified in *Lactobacillus helveticus* (Ojennus et al., 2019) and *Lactococcus lactis* (Nurizzo et al., 2002) could hydrolyze peptide bonds from the N‐terminus of substrates when the penultimate amino acid residue is a proline. The highly induced dipeptidyl‐peptidase might suggest the high content of oligopeptides with penultimate proline residue in the fermented soymilk. In the stationary phase, there were only two aminopeptidase genes (*pepC*, B1745_00910 and B1745_01515) that were significantly up‐regulated, which might be due to the stagnation of cell growth and proliferation, reducing the requirement for peptide and amino acid.

Genome analysis of *L. amylolyticus* L6 with KEGG pathways revealed that this strain was able to synthesize nine kinds of amino acids, including valine (Val), leucine (Leu), isoleucine (Ile), phenylalanine (Phe), tryptophan (Trp), tyrosine (Tyr), aspartate (Asp), asparagine (Asn), and arginine (Arg). *asnA* (B1745_06855) and *lysC* (B1745_RS07280) genes responsible for the synthesis of aspartate (Asp) showed high induction levels in the logarithmic phase (Table 2), while other amino acid synthetic genes were not up‐regulated. Meanwhile, the content of asparagine in the fermented soymilk reduced significantly from 21.7 mg/L to 5.48 mg/L, and this phenomenon did not occur in other amino acids (Table 4). The data presented in this study suggested that the growth and proliferation of *L. amylolyticus* L6 required a large amount of aspartate and asparagine, therefore promoting the uptake of free asparagine from soymilk into cell and simultaneously synthesizing aspartate by inducing the expression of *asnA* and *lysC*. In the stationary phase, *asnB* gene that encoded asparagine synthase (glutamine‐hydrolyzing) catalyzing the conversion of aspartate into asparagine with glutamine as the nitrogen source was significantly down‐regulated, which was due to the feedback repression by a high concentration of substrate glutamate inside and outside the cell.

*L. amylolyticus* L6 could not synthesize other amino acids, except those mentioned above, hence it needed the help of amino acid transporters to supplement amino acid. A glutamate transporter operon (glnQHMP, *glnP*, B1745_05185; *glnM*, B1745_05190; *glnH*, B1745_05195; *glnH*, B1745_05195) was highly induced in the logarithmic phase to transport the high concentration of free glutamate from the soymilk into the cell (Table 2 and Table 4). The overexpression of the glutamate transporter operon has also been reported in *L. casei* Zhang under soymilk environment (Wang et al., 2012). Meanwhile, many uncharacterized amino acid permease genes (B1745_03105, B1745_06875, B1745_06870, and B1745_06860) were up‐regulated, while two amino acid permease genes (B1745_04680 and B1745_03815) were down‐regulated in the logarithmic and stationary phase. Interestingly, two genes *livB* and *brnQ* coding for branched‐chain amino acid transport system II carrier protein and branched‐chain amino acid ABC transporter permease were significantly down‐regulated in the stationary phase. That's because *L*. *amylolyticus* L6 could synthesize branched‐chain amino acids (leucine, isoleucine, and valine) and did not require the help of their transporters, therefore repressing the expression of corresponding genes.

### 3.5 Lipid metabolism, inorganic ion transport, and stress response

There are 14 genes involved in fatty acid biosynthesis which were identified in the genome of *L*. *amylolyticus* L6, which includes *accA* (acetyl‐CoA (coenzyme A) carboxylase), *accB* (acetyl‐CoA carboxylase biotin carboxyl carrier protein), *accC* (acetyl‐CoA carboxylase, biotin carboxylase subunit), *accD* (acetyl‐CoA carboxylase carboxyl transferase subunit beta), *fabD* (acyl‐carrier‐protein S‐malonyltransferase), *fabF* (3‐oxoacyl‐[acyl‐carrier‐protein] synthase II), *fabG* (3‐oxoacyl‐acyl‐carrier protein reductase), *fabH* (3‐oxoacyl‐[acyl‐carrier‐protein] synthase III), *fabI* (enoyl‐[acyl‐carrier protein] reductase I), *fabZ* (3‐hydroxyacyl‐[acyl‐carrier‐protein] dehydratase), and *mch* (medium‐chain acyl‐[acyl‐carrier‐protein] hydrolase). Only *accC* gene (B1745_05670) was found to be up‐regulated in the logarithmic phase during the growth of *L*. *amylolyticus* L6 in soymilk. It was reported that soymilk contained 2.64% grease, and the relative content of unsaturated fatty acid is more than 80% (Xiangnan et al., 2019), which inhibited the expression of genes related to fatty acid biosynthesis in *L*. *amylolyticus* L6. Meanwhile, acyl‐CoA thioesterase gene (B1745_02830) that catalyzes the hydrolysis of acyl‐CoAs to the free fatty acid and regulates intracellular levels of free fatty acids and acyl‐CoAs (Tillander et al., 2017) was highly induced during its growth in soymilk. Besides, several genes coding for esterase (B1745_05970 and B1745_00245) were also up‐regulated in logarithmic phase to utilize the grease in soymilk.

Inorganic ions, especially metal ions, are important for LAB to maintain normal functions in the metabolism (Mrvčić et al., 2012). Generally, membrane transporters play a crucial role in regulating the intracellular concentrations of metal ions (Boyaval, 1989). The expression of five genes, such as *mgtC* (B1745_00100) coding for Mg^2+^ transporter, *pot* (B1745_00845) coding for potassium transporter, *amt* (B1745_05305) coding for ammonium transporter, and cadmium‐translocating P‐type ATPase gene (B1745_06945), was significantly induced in the logarithmic phase, indicating the importance of inorganic ions in regulating physiological functions of *L. amylolyticus* L6, such as ion homeostasis, coenzyme factor, and electron transport system.

During the fermentation, the pH values and acidity of soymilk in the stationary phase could reach 4.0 and 95.88°, respectively, which would induce the expression of genes in responding to acidity stress. Molecular chaperones have been regarded as a ubiquitous feature of cells, including LAB, in which these proteins cope with stress‐induced denaturation of other proteins (Feder & Hofmann, 1999). Chaperone proteins GroL, DnaK, and GrpE participate actively in the response to stress conditions by preventing the aggregation of stress‐denatured proteins (Lemos et al., 2007). Transcriptomic analysis indicated that the expression of genes *groEL* (B1745_01775), *dnaK* (B1745_03015), and *grpE* (B1745_03010) coding for chaperone proteins was highly up‐regulated in the stationary phase, while these two genes were not significantly induced in logarithmic phase. The difference was mainly due to a relatively higher pH value in logarithmic phase that is not enough to cause acid stress to *L. amylolyticus* L6 (Table 1). The increased expression level of a universal stress protein (B1745_00745) in the stationary phase that was required for resistance to DNA damage also engaged in acid tolerance of *L. amylolyticus* L6. In addition, the high transcript level of thioredoxin (*trxA*, B1745_01850) in the stationary phase that acts as an antioxidant by promoting the reduction of other proteins through the cysteine thiol–disulfide bond exchange was related to stress adaptation in *L. amylolyticus* L6. The gene highly expressed in logarithmic phase was glutamate:gamma‐aminobutyrate antiporter (*gadC*, B1745_00320) that exchanges the intracellular γ‐aminobutyric acid (GABA) with extracellular Glu to expel protons in the cytoplasm (Dan et al., 2012).

### 3.6 Change of isoflavones in fermented soymilk

Soymilk was rich in isoflavonesi in the form of isoflavone aglycones (10%) and their corresponding glucosidic conjugates (90%) (Rodriguez‐Roque et al., 2013). Isoflavones’ glucosidic conjugates could be converted into highly bioactive aglycones by *β*‐glucosidase in lactobacilli (Tang et al., 2007; Wei et al., 2007; Xia et al., 2019). As shown in Table 5, most of the isoflavones in unfermented soymilk occurred in the form of glucosides with the concentration of 285.77 mg/L and the content of aglycones was only 14.51 mg/L. During the fermentation, the total concentration of isoflavone aglycones increased from 14.51 mg/L to 36.09 mg/L, and three forms of aglycones’ (daizein, glycitein, and genistein) concentration also increased significantly. However, the content of glucosidic isoflavones changed irregularly during the fermentation. Compared with the unfermented phase, the glucosidic isoflavones (daidzin, glyctin, and genistin) exhibited a decreasing tendency in the lag phase (2h), and then the content of glucosidic isoflavones increased gradually in the logarithmic and stationary phase. A similar phenomenon has been reported in the soymilk beverage fermented by Kombucha rich in LAB (Xia et al., 2019). It is presumed that the fermentation of *L. amylolyticus* L6 could promote the release of free flavonoids from binding forms with soluble fibers in the soymilk. Transcriptomic data indicated that the expression of *bglA* gene coding for 6‐phospho‐*β*‐glucosidase increased significantly in logarithmic phase, which was consistent with the increasing concentrations of isoflavone aglycones. And 6‐phospho‐*β*‐glucosidase that could convert isoflavone glucosides into aglycones has been reported in our previous study (Fei, Liu, et al., 2017).

**TABLE 5:** Concentration of isoflavones (mg/L) in soymilk fermented with *L*. *amylolyticus* L6

Isoflavones (mg/L)	Period			
	Unfermented	Lag phase	Log phase	Stationary phase
Glycosides				
Daidzin	216.65 ± 3.80^a^	154.96 ± 1.92^c^	188.45 ± 1.83^b^	217.22 ± 2.01^a^
Glyctin	46.02 ± 1.77^b^	35.17 ± 0.47^d^	43.60 ± 0.19^c^	50.73 ± 0.29^a^
Genistin	23.09 ± 0.12^a^	11.87 ± 0.63^b^	12.10 ± 0.06^b^	12.62 ± 0.77^b^
Total	285.77 ± 5.50a	202.01 ± 1.80c	244.15 ± 1.87b	280.57 ± 1.41a
Aglycones				
Daizein	10.03 ± 0.49^c^	9.17 ± 0.48^d^	11.89 ± 0.08^b^	16.63 ± 0.14^a^
Glycitein	ND	ND	ND	5.61 ± 0.07^a^
Genistein	4.48 ± 1.16^b^	5.65 ± 1.87^b^	12.11 ± 0.99^a^	13.85 ± 1.27^a^
Total	14.51 ± 1.65 ^c^	14.82 ± 0.62 ^c^	24.00 ± 1.07 ^b^	36.09 ± 1.48 ^a^

Note

Data are the mean±standard deviation (*n* = 3). Means in the same column with different superscript letters (a–d) are significantly different (*p* <.05). ND means not detected.

## 4 CONCLUSION

This study revealed the chemical component changes and transcriptomic changes of *L*. *amylolyticus* L6 in fermented soymilk. Large amount of genes related to carbon metabolism in *L*. *amylolyticus* L6 were significantly up‐regulated in the logarithmic phase and stationary phase, which allowed this strain to metabolize various sugars in soymilk. Highly expressed α‐galactosidase gene could help to reduce the content of raffinose and stachyose that caused flatulence of human body. Meanwhile, the concentration of total amino acid increased significantly in the logarithmic phase for highly induced genes involved in the proteolysis, hydrolysis, and transport of peptide, transport and biosynthesis of amino acid. Highly efficient utilization of carbon and nitrogen sources significantly raised the viable counts of *L. amylolyticus* L6 in soymilk. High expression of 6‐phospho‐*β*‐glucosidase promoted the conversion of isoflavone glycoside into highly bioactive aglycones. Besides, other genes related to lipid metabolism, inorganic ion transport, and stress response were also up‐regulated. Further study should be conducted in terms of applying this strain into developing soymilk products and vitro digestion simulation test to testify its production performance. In conclusion, this study reveals that *L. amylolyticus* L6 isolated from the soybean‐derived environment exhibited excellent adaptability in a soymilk‐based ecosystem, which is expected to become the specific probiotic strain used for the fermentation of soybean products.

## CONFLICT OF INTEREST

None declared.

## ETHICAL APPROVAL

The authors declare that they have no conflict of interest. This article does not contain any studies involving animal's trails performed by any of the authors. Furthermore, this article does not contain any studies involving human participants performed by any of the authors.

## Supporting information

Supplementary Material

Supplementary Material

## Data Availability

The original contributions presented in the study are included in the article/Supplementary Material, and further inquiries can be directed to the corresponding authors.
