# Supplementary figures and images for: Adaptive mechanism of Lactobacillus amylolyticus L6 in soymilk environment based on metabolism of nutrients and related gene‐expression profiles
Source: Food Sci Nutr. 2022 Feb 23;10(5):1548–63. doi: 10.1002/fsn3.2779 (PMC9094474; doi:10.1002/fsn3.2779)

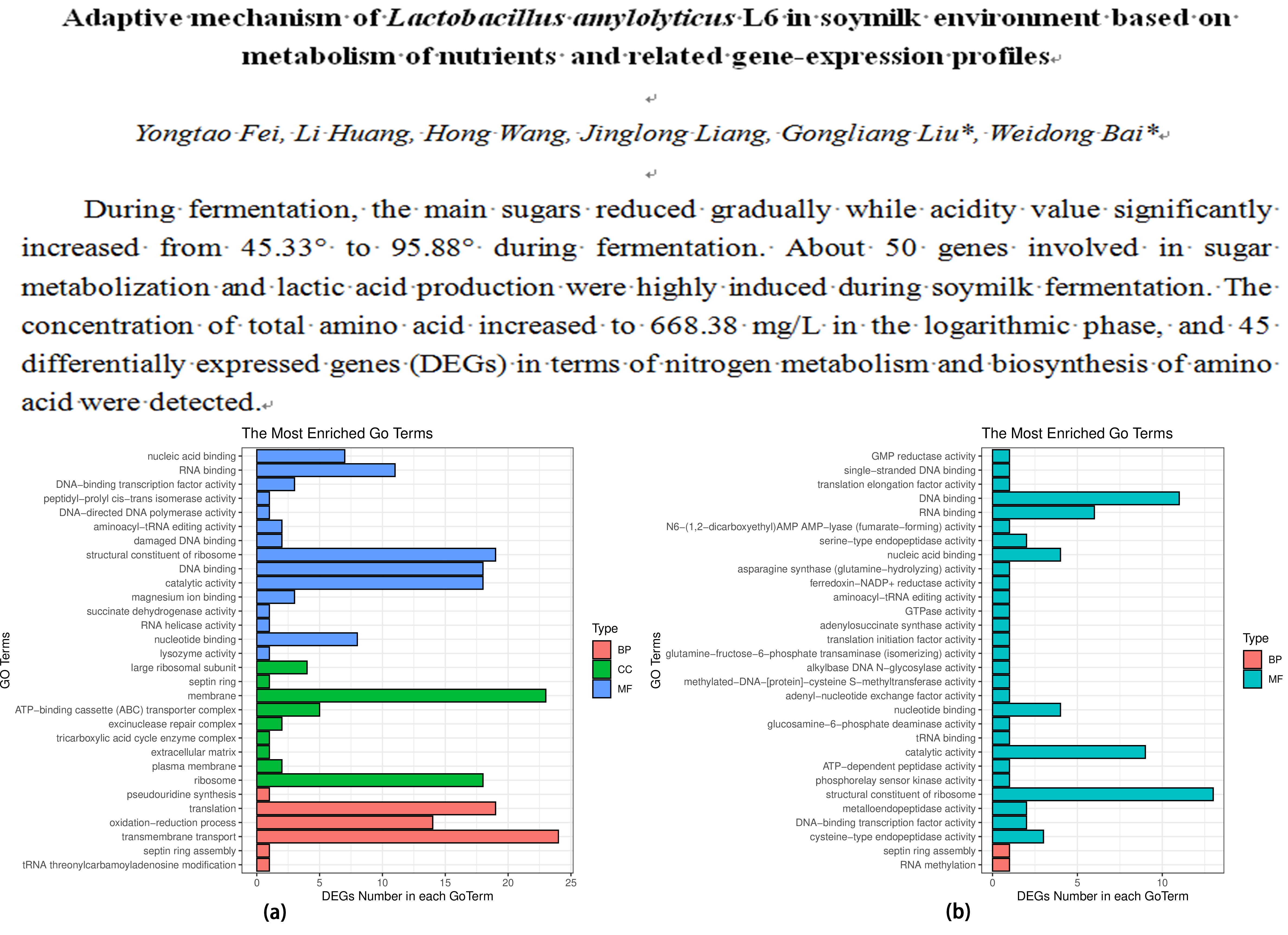

Supplement: Supplementary file 1 — Supplementary Material [file FSN3-10-1548-s001.jpg]
