# Supplementary material for: Adaptive mechanism of Lactobacillus amylolyticus L6 in soymilk environment based on metabolism of nutrients and related gene‐expression profiles
Source: Food Sci Nutr. 2022 Feb 23;10(5):1548–63. doi: 10.1002/fsn3.2779 (PMC9094474; doi:10.1002/fsn3.2779)

**Table S1**

**Primers used for RT-qPCR**

| Locus | Primer | Sequence(5’-3’) |
| --- | --- | --- |
| B1745_04860 | F | TCGCTGTTTTCGCCGTTAAC |
|  | R | TTCGTCCCAGTTGAAATCGC |
| B1745_00910 | F | AACCAGAAGCAGTCAGGACG |
|  | R | GTTGGCGCGTTCAATTCTGT |
| B1745_00925 | F | TTGCCGGCAACTTTGAGATC |
|  | R | AAGCAGCAACCAACTTGTCG |
| B1745_01705 | F | ATTGCGGATTTGCTCAAGCG |
|  | R | TACATGTTCGCCTGGTTTGG |
| B1745_01760 | F | TTGGGCTTGGCAGTTAAACC |
|  | R | TTTTCATCGCGGTGGTTTCC |
| B1745_01765 | F | ATCAGCTGGTGTTTCTGCAC |
|  | R | AAGCAGCAGCAGTAACATGC |
| B1745_02440 | F | AGAAAGCACGGTTTGTTCGC |
|  | R | ACATGTTGGTGTGCATTCCG |
| B1745_03115 | F | AAGACTTTTGCCAGCTTGCC |
|  | R | GACTGAAACTTGCGGATTGTCC |
| B1745_03165 | F | TGTTTCCGCTAACCCAGTTG |
|  | R | TTTGCAACAGGAACGCCAAC |
| B1745_03855 | F | TTCAGCAACCAAACCACGTG |
|  | R | TGAAGCCCATTTCAGCCATC |
| B1745_05130 | F | AAAATGGCTACTGGGTTGGC |
|  | R | GTGTTTGGCAAAGTGGTTGG |
| B1745_06165 | F | AAAGCAACGCGTAACTCAGC |
|  | R | TGCTGCTCTTGCTTTTGGAC |
| B1745_06755 | F | AAGAAGGGCAATGCTCAAGC |
|  | R | TCTTGCCGCCAACTTCAATC |
| B1745_07025 | F | TGCTTGCGGAAGATATTGCG |
|  | R | AAATCCGTCTGACGAATGCG |
| B1745_08070 | F | ATGTGTCAGTCAGCCCAAAC |
|  | R | AAATCGCCCCACATTGCAAC |
| Reference Gene | 16S-F | ACTCCTACGGGRSGCAGCAG |
|  | 16S-R | ATTACCGCGGYKGCTGG |

**Fig.S1. Statistics of SDGEs among different growth phase**


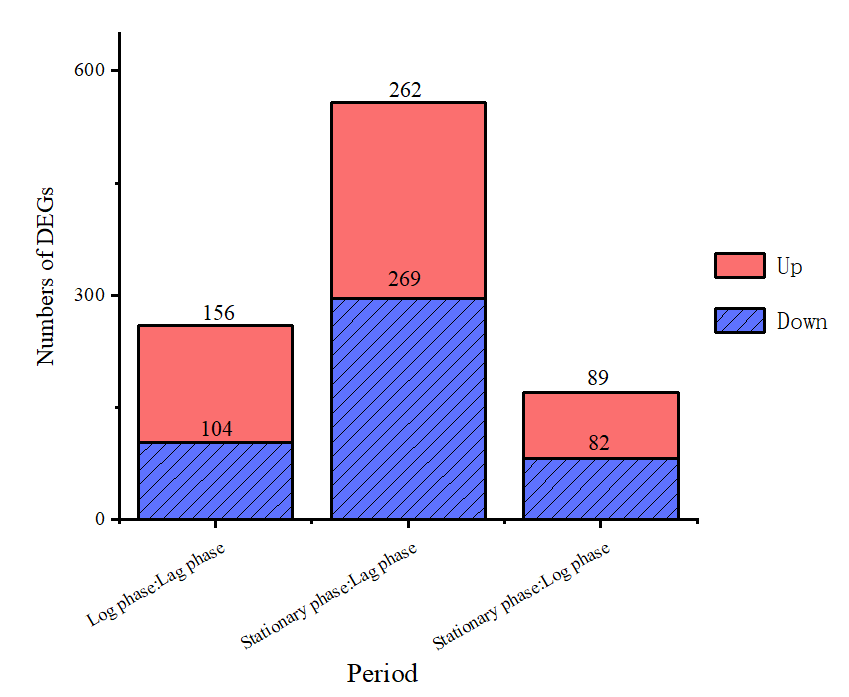

Supplement: Supplementary file 2 — Supplementary Material [file FSN3-10-1548-s002.docx]
